# Lexical-Semantic Development in Bilingual Toddlers at 18 and 24 Months

**DOI:** 10.3389/fpsyg.2020.508363

**Published:** 2020-12-17

**Authors:** Stephanie De Anda, Margaret Friend

**Affiliations:** ^1^Department of Special Education and Clinical Sciences, University of Oregon, Eugene, OR, United States; ^2^Department of Psychology, San Diego State University, San Diego, CA, United States

**Keywords:** bilingual, word recognition, toddlers, translation equivalents, vocabulary, Spanish

## Abstract

An important question in early bilingual first language acquisition concerns the development of lexical-semantic associations within and across two languages. The present study investigates the earliest emergence of lexical-semantic priming at 18 and 24 months in Spanish-English bilinguals (*N* = 32) and its relation to vocabulary knowledge within and across languages. Results indicate a remarkably similar pattern of development between monolingual and bilingual children, such that lexical-semantic development begins at 18 months and strengthens by 24 months. Further, measures of cross-language lexical knowledge are stronger predictors of children’s lexical-semantic processing skill than measures that capture single-language knowledge only. This suggests that children make use of both languages when processing semantic information. Together these findings inform the understanding of the relation between lexical-semantic breadth and organization in the context of dual language learners in early development.

## Introduction

Research in early language acquisition examines developmental changes in lexical growth. This body of work reveals rapid word learning during the first and second year of life, such that young infants begin babbling early in the first year (e.g., Molemans et al., 2012). Soon, young children approximate words and say their first word around 12 months. By the end of the second year at 24 months, children begin producing their first word combinations. Importantly, these language milestones are met at the same time for children learning one or two languages (Pearson et al., 1993; Pearson and Fernández, 1994). Indeed, monolinguals and bilinguals use similar word learning strategies and learn words at a similar rate overall (Pearson et al., 1993; Byers-Heinlein et al., 2013). Further, recent evidence has shown that bilingual and monolingual toddlers access semantic information in single words at a similar rate at 16 and 22 months (De Anda et al., 2018; Legacy et al., 2018).

Despite several similarities between monolingual and bilingual toddlers in word acquisition and processing, it is unclear how dual language exposure influences lexical organization. In large part, studies of lexical development have focused on the acquisition of individual words. Few studies have examined how words and their meanings are semantically organized to support spoken word processing and fewer still have studied this in dual language contexts in young learners. At present there remains a dearth of studies investigating the emergence of lexical networks in bilingual children to parallel that in the monolingual literature. Indeed, there is reason to believe that lexical-semantic organization specifically may differ between bilingual and monolingual toddlers based on theoretical and empirical work. Bilingual children are tasked with organizing words in a lexicon to encompass two languages. Although it is unclear whether bilingual lexical-semantic networks are comprised of shared or separate representations across languages, it is clear that lexical processing occurs in parallel across languages in bilingual adults and in bilingual toddlers as early as 30 months of age (Kroll et al., 2010; Singh, 2014). Nevertheless, the emergence of this shared processing in dual language contexts is not well understood. Indeed, recent calls have been made to shift our research focus to lexical organization across single and dual language learners (Wojcik, 2017). We review extant findings and introduce testable predictions about the emergence of lexical-semantic organization in bilingual first language acquisition below.

In a handful of studies, researchers have used lexical-semantic priming tasks to assess lexical networks in young infants and toddlers. Within monolinguals, findings reveal an incremental developmental process, in which lexical-semantic priming emerges late in the second year around 21 months of age in English learners (for a review, see De Anda et al., 2016a). Specifically, 21-month-old, but not 18-month-old, English monolinguals showed longer looking to the target object when it was preceded by a semantically related word relative to trials in which it was preceded by a semantically unrelated word, thereby indicating that lexical-semantic priming effects emerge around 21 months of age (Arias-Trejo and Plunkett, 2009). Further, 21-month-old monolinguals exhibited priming between words that were associatively *and* semantically related. By 24 months, however, either an associative *or* a semantic relation was sufficient to elicit priming (Arias-Trejo and Plunkett, 2013). Together these findings suggest the fragile emergence of lexical-semantic structure by 21 months of age that becomes more robust by the end of the second year at 24 months in monolinguals. Of interest in the present study is the influence of dual language exposure on this developmental timeline and the role of lexical knowledge in within and across languages in supporting this emergence. Specifically, we ask whether bilinguals exhibit lexical-semantic priming as early as 18 and 24 months of age and whether this processing skill is associated with lexical knowledge.

To date, few studies have investigated lexical priming in bilingual contexts. Extending previous work in monolinguals, Von Holzen and Mani (2012) examined whether bilinguals’ second language (L2) primed the first language (L1) in phonologically and semantically related word pairs between 21 and 43 months of age. Remarkably, bilingual toddlers showed facilitated target recognition, even in cases where phonological priming occurred indirectly through a translation equivalent (TE). For example, children showed facilitated recognition of the L1 German target “stein” given the L2 English prime of “leg.” This priming relation appeared to be supported by the phonological overlap between the L1 target (“stein”) and the L1 semantic translation of the L2 prime, “bein.” This finding demonstrates two key points. First, German-English bilinguals activate phonological knowledge from L2 to L1. Second, like monolinguals, bilinguals exhibit implicit activation of TEs when processing in L2 after age 2. These results parallel the findings and theoretical accounts of adult bilingual language representation given that cross-language phono-semantic co-activation extends, at least in this case, to early childhood. Further, they suggest that shared lexical-semantic knowledge across languages (as in TEs) supports priming.

Of particular interest in the present study is the development of semantic associations in dual language contexts. In a study of 30-month-old Chinese-English bilinguals, Singh (2014) investigated bidirectional lexical-semantic priming within and across the dominant and less-dominant language. Within-language priming was observed for the dominant, but not the non-dominant, language. Further, cross-language priming was unidirectional, such that the dominant language primed the non-dominant language, but the opposite was not true. Put another way, in contrast to Von Holzen and Mani (2012), priming was only observed when the semantic prime was in the dominant language. That is, bilinguals show priming from the non-dominant to the dominant language when *both* phonological and semantic information are provided in the third year of life as in Von Holzen and Mani (2012). However, given only semantic information, 30-months-old show priming effects only when the prime occurs in the dominant language (Singh, 2014). Similar results have recently been reported in Spanish-English dual language learning children at 7.5 years of age (Goodrich and Lonigan, 2018) and in French-English learners at 30 months of age (Jardak and Byers-Heinlein, 2018).

Together, previous findings support the existence of a shared lexical-semantic network in bilingual first language acquisition. Within lexical-semantic studies, the findings by Singh (2014) suggest connections between semantically related words are observed in the second year similar to monolingual children. Nevertheless, there remain gaps in our understanding of the *emergence* of lexical priming in bilingual development before age 2 to parallel work in monolinguals. As discussed previously, the monolingual literature indicates a fragile emergence of lexical-semantic priming by 21 months of age with more robust effects found after the second birthday. One key limitation in previous studies of bilingual lexical-semantic priming is the focus on total proportion looks to the target as the dependent measure and underpowered studies with small sample sizes (Singh, 2014; Jardak and Byers-Heinlein, 2018). This relatively coarse measure of language processing collapses gaze responses over the entire trial. Though these analyses are powerful for detecting differences across a time window of interest, changes in looking behavior as a function of time are not captured. Conversely, at a finer level of analysis, it may be possible to detect emergent semantic organization at or prior to 24 months in bilingual children by examining behavior at a millisecond-by-millisecond level. We propose to address this limitation in two ways. First, we conduct a-priori power analyses to ensure a sufficient sample size to detect coarse-level differences in looking as a function of lexical-semantic associations. Second, we incorporate time-course analysis to detect the potentially fragile emergence of lexical-semantic organization during the second year in bilingual toddlers. Indeed, neurophysiological findings suggest distinct lexical-semantic processing patterns in the dominant vs. the non-dominant language by 24 months of age (Conboy and Mills, 2006; Sirri and Rämä, 2015). We outline our predictions about the developmental time course and emergence of lexical-semantic networks in dual language learning contexts by 24 months of age below.

Do bilinguals begin to form lexical-semantic connections between languages, and therefore begin to process both languages in parallel, at the same time that monolinguals form connections between words in their single language? Is the development of lexical-semantic priming a robust process that emerges similarly across single and dual language learners? And how does vocabulary knowledge within and across languages support lexical-semantic organization? One account suggests that lexical-semantic connections between and within languages arise at the same age for monolingual and bilingual children. Indeed, many models of early language acquisition consider the end of the second year to be an important time for lexical development. For example, researchers have documented an acceleration in vocabulary size that occurs at approximately 18 months of age across bilingual and monolingual children (Goldfield and Reznick, 1990; Pearson et al., 1993; McMurray, 2007; McMurray et al., 2012). This rapid growth in the lexicon might have implications for lexical organization; object categorization might foster connections between related words within and across languages. Thus, under a maturational account, the acceleration in lexical acquisition around age 2 gives rise to the emergence of lexical networks independent of the number of languages being learned.

A second possibility is that the emergence of lexical organization occurs earlier for bilinguals relative to monolinguals. There are several aspects of bilingual language acquisition that support the notion of precocious development of the lexical-semantic system. That is, it may be that the task of forming connections between two semantically related non-TEs across languages (such as *dog* and *gato* in the context of the present study) within a network might be easier, and perhaps develop earlier for bilinguals. Bilinguals might be cued into the connections between words given their unique experience and lexical knowledge, namely with cognates and TEs. That is, cognates and TEs have a *compound* structure (De Groot, 1993): two separate lexemes are bound to a single concept, thereby forming an indirect connection between two lexemes. As we review below, this enhanced compound lexical structure in bilinguals relative to monolingual learners has implications for language learning and word retrieval and may also influence lexical-semantic organization.

One proxy of compound lexical structure in bilinguals is the number of known TEs. Byers-Heinlein and Werker (2013) found that 17-month-old bilinguals, in contrast to monolinguals, are more likely to accept a novel name for a previously named object. This propensity varies as a function of the number of TEs known across languages: bilinguals who knew many TEs were more likely to show this effect relative to bilinguals who knew fewer TEs. The authors propose the *lexicon structure hypothesis*, which suggests that bilingual infants with knowledge of many TEs have richer semantic organization relative to monolingual peers, as the relationship between words and concepts across two languages supports a many-to-one mapping structure. Similarly, Poulin-Dubois et al. (2013) found that bilinguals with many TEs show faster lexical access than bilinguals with fewer TEs, suggesting a facilitative priming effect that follows from a many-to-one lexical-semantic organization. Extending these findings to semantic relations in the lexicon, it is possible that bilinguals might be cued into the relationship between two semantically associated non-TEs across languages before age 2. That is, they might form connections between *dog* and *gato* before monolingual children form relations between *dog* and *cat* due to a rich and complex lexical network that is leveraged across languages. Further, measures of cross-language lexical knowledge, such as number of known TEs, may predict the magnitude of children’s lexical-semantic processing skill.

A final possibility is that bilinguals might show later emergence of lexical networks than their monolingual peers. This would be consistent with the *resource limitation hypothesis*, which suggests that bilinguals face more challenges in word learning relative to monolinguals in using phonetic detail, for example (Stager and Werker, 1997; Werker et al., 2002; Fennell and Werker, 2003; Werker and Fennell, 2004; Fennell et al., 2007). Despite being exposed to greater phonetic breadth than monolinguals, bilinguals may have weaker phonemic representations by virtue of having relatively less exposure, since exposure is split across languages. Although bilinguals learn words at the same rate as monolinguals (Pearson et al., 1993), monolinguals seem to utilize phonemic detail to guide word learning earlier in development. Thus, it is possible that these weak phonemic representations might lead to weaker connections between words, at least at the phonological level. Whether this account extends to the semantic domain remains to be examined.

Extending the Distributed Feature Model (DFM, Van Hell and De Groot, 1998) to early development also suggests the possibility of a protracted account of semantic connections within and across languages for bilinguals. The DFM characterizes lexical-semantic connections as highly contextualized, thereby accounting for subtle differences in meaning in TEs, a unique product of the bilingual lexicon. In monolinguals, the DFM would account for the emergence of semantic priming around age 2 as a result of strengthened semantic representations that activate words with similar meaning. That is, as children encounter more exemplars of *dog* and *cat*, they activate an increasing number of overlapping semantic features that lead to the semantic priming effects observed in the second year of life. Yet, bilinguals’ experience with the world is split between two languages. That is, bilinguals may receive half as many exemplars per lexical item relative to monolinguals, which may lead to weaker semantic representations of single words if lexical-semantic systems are built relatively independently in early development. In turn, these semantic representations make associations (and priming) across words less probable. If the emergence of semantic priming in monolinguals at age 2 is driven by amount of semantic overlap between words, then bilinguals may have a more protracted development of semantic connections, as there are fewer overlapping semantic features for related lexical items. This account predicts slower and later-emerging lexical-semantic priming for monolinguals relative to bilinguals.

The present study seeks to examine lexical-semantic development as it emerges during the second year. Specifically, we ask: When do lexical-semantic priming effects emerge over the second year in simultaneous bilingual toddlers? To accomplish this, we ensured that an adequate level of power was reached based on previously reported effect sizes. Further, we developed an analytic strategy that encompasses both coarse‐ and fine-grained levels of analysis to detect fragile moment-by-moment changes in gaze behavior that both replicates and extends previous work. Lastly, we examine lexical-semantic priming longitudinally at 18 and 24 months of age in Spanish-English bilinguals to ensure that the earliest emergence of semantic organization are captured. As we have reviewed, the dearth of research in this area leaves several possible predictions open with respect to the development of the lexical-semantic priming during the second year in the context of dual language learners.

**Figure 1:**
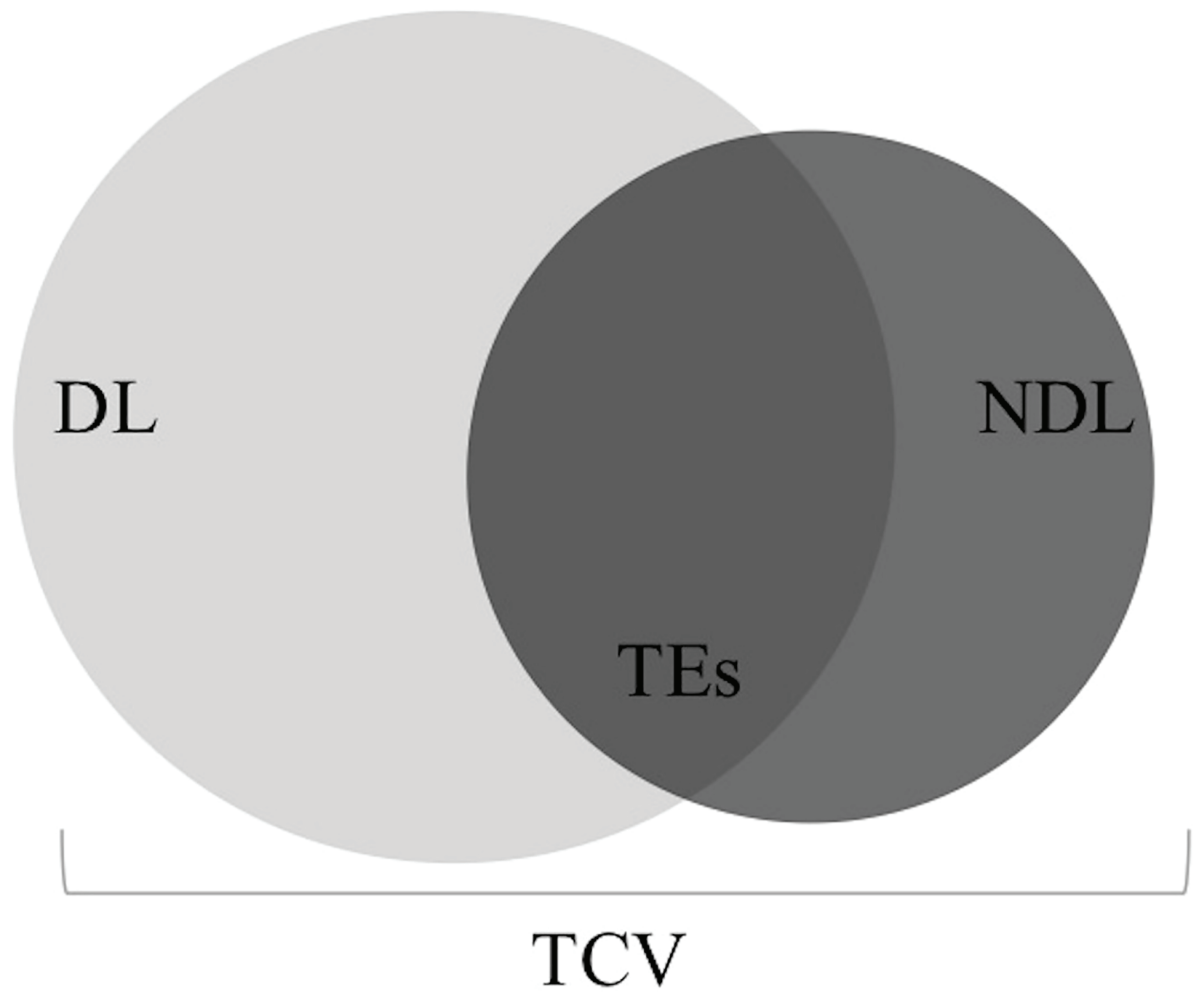
Model of overlap between lexical-semantic space across the dominant and non-dominant language underlying priming effects. DL = Dominant Language vocabulary, NDL = Non-Dominant Language vocabulary, TEs = Translation Equivalents, and TCV = Total Conceptual Vocabulary.

The second research question examines the unique features of bilingual children’s lexical knowledge to investigate their relation to children’s lexical-semantic processing skills. Indeed, lexical-semantic structure in early bilingual first language acquisition encompasses within-language vocabulary in the dominant and non-dominant language, as well as cross-language knowledge such as with TEs and total vocabulary (Figure 1). It is possible that these unique lexical contexts may lead to differences in the emergence of lexical-semantic priming for children with dual language exposure relative to their monolingual counterparts as discussed previously. Yet, even if lexical-semantic priming emerges at similar ages for monolingual and bilingual children, it is possible that the underlying sources of variability differ, especially given that bilinguals construct dual lexicons unlike monolinguals. Therefore, the present longitudinal study examines vocabulary and lexical-semantic priming in Spanish-English bilingual toddlers at 18 and 24 months of age. Together these research questions begin to uncover the developmental time course of lexical-semantic processing and the sources of variability that support its emergence.

## Materials and Methods

### Participants

A total of 32 bilingual English‐ and Spanish-learning toddlers (15 females and 17 males) participated in the present longitudinal study at 18-months (*M* = 18;17, range = 17;15–20;21) and 24-months of age (*M* = 25;5, range = 23;6–27;12). Seven participants from the original sample of 32 did not return for the 24-month testing occasion. We nevertheless analyzed all of the available data within each age group. An *a priori* power analysis (Faul et al., 2007) was conducted to determine the appropriate sample size based on the smallest effect size reported in a similar study (Arias-Trejo and Plunkett, 2009). Results indicated that a sample size of at least 20 participants was required to achieve appropriate power (1-β > 0.9). It was important to ensure adequate power was reached because of the interest in evaluating the fragile emergence of lexical-semantic priming effects during the second year in the present study. This was especially important in the context of coarse-level looking time measures (e.g., proportion of total looks to the garget) as small changes in looking behavior may go undetected when collapsing data over time.

The average maternal education was approximately completion of a 4-year college degree (*M* = 14.97 years, *SD* = 2.27, range = 11–18). Participants were obtained through a database of parent volunteers recruited through birth records, internet resources, community organizations, and events in a large metropolitan area in California. All participants were born at full-term and had no diagnosed impairments in hearing, vision, language, and cognition per parent report. To be included in the present study, children must have had between 20 and 80% exposure to both English and Spanish as determined by the Language Exposure Assessment Tool (LEAT, DeAnda et al., 2016b). This range is based on several studies of early simultaneous bilinguals which together show that exposure greater than 20% supports language use (Pearson et al., 1997; Gutiérrez-Clellen and Kreiter, 2003; Fiestas and Peña, 2004; Friend et al., 2018). No children had exposure to a third language. All children received early simultaneous exposure to both English and Spanish. The study was carried out in accordance with the recommendations of the National Institutes of Health Guidelines for the Review of Human Subjects. The protocol was approved by the Institutional Review Board at *removed for blinding* University. All subjects gave written informed consent in accordance with the Declaration of Helsinki.

### Measures

#### The Computerized Comprehension Test

The computerized comprehension test (CCT) is a behavioral measure that captures children’s haptic response to assess early decontextualized receptive vocabulary. The CCT converges with parent report on the MacArthur-Bates Communicative Development Inventory (MCDI, Fenson et al., 2006), and predicts subsequent language production (Friend et al., 2012). Additionally, responses on the task are nonrandom across languages (Friend and Zesiger, 2011) and across monolinguals and bilinguals (Poulin-Dubois et al., 2013). The English and Spanish CCT have good test-retest reliability (*r* = 0.7 and 0.76 in English and Spanish, respectively; Friend et al., 2018) and demonstrate strong internal consistency (Cronbach’s α; English = 0.91 form A and 0.95 form B; Spanish = 0.77 form A and 0.91 form B; De Anda et al., 2016a).

The procedures and construction of the CCT are identical across the English and Spanish adaptations with the exception that items for each adaptation were chosen based on their age of acquisition (see below). Participants are prompted to touch images on the monitor following systematic sentence prompts based on the target (Noun prompts: “Where is the [target]? Touch [target?. / Donde esta el/la [target]? Toca [target].; Verb prompts: Who is [target]? Touch [target]. / Quien esta [target]? Toca [target]. Adjective prompts: Which one is [target]? Touch [target]. / Cual es [target]? Toca [target]). There are four training trials and 41 test trials in a two-alternative forced-choice procedure. For each trial, two images (a target and distractor image) appear simultaneously on the right and left side of the touch monitor. The side of the target image occurred in pseudo-random order across trials such that target images could not appear on the same side on more than two consecutive trials, and the target was presented with equal frequency on both sides of the screen. There is an equal representation of easy, medium, and difficult words. All verbs are human actions (e.g., kissing and playing) and adjectives are colors (e.g., orange and red) or states (e.g., happy, old, and full). All image pairs are matched for word difficulty (easy, moderate, and difficult) based on MCDI and Inventario de Desarrollo de Habilidades Comunicativas (IDHC) norms (Fenson et al., 2007; Jackson-Maldonado et al., 2003; Frank et al., 2017), part of speech (noun, adjective, and verb), and visual salience (color, size, and luminance). Further, nouns are matched on category (animal names, vehicles, toys, body parts, food and drink, and household objects), whereas adjectives were matched on type (color or state; Friend and Keplinger, 2008). Target words were distributed over 23 nouns, 11 verbs, and seven adjectives. Of the nouns, seven were animals, three were vehicles, two were toys, three were body parts, three were food and drink, and five were household objects in English, and six were animals, one was a toy, two were body parts, four were food and drink, and nine were objects in Spanish.

The CCT begins with a training phase to ensure participants understand the nature of the task. During the training phase, participants were presented with early-acquired noun pairs (known by at least 80% of 16-months-old; Frank et al., 2017) and prompted by the experimenter to touch the target. If the child failed to touch or unambiguously point to either image on the screen after repeated prompts, the experimenter touched the target image for them. If a participant failed to touch, the training trials were repeated once. All participants executed at least one correct touch during the training phase and proceeded to the test phase.

During the test phase, each test trial ended when the child touched the screen or until 7 s elapsed. When child gaze was directed toward the touch monitor, the experimenter delivered the prompt in infant-directed speech and advanced each trial. The experimenter presented each pair of images as she uttered the target word in the first sentence prompt such that the onset of the target word occurred just prior to the onset of the visual stimuli. If the child failed to provide a response for three consecutive trials, all three trials would be coded as “no response” and the experimenter modeled a correct touch (as in the training phase) before moving on to the next trial. Accuracy on the Spanish and English CCT test trials provided a behavioral measure of children’s within-language receptive vocabulary size.

#### The MacArthur Bates Communicative Development Inventory

The MCDI is a widely used parent report measure of early language. The Words and Gestures inventory is a checklist on which parents mark the words their child understands and says. The inventory provides an indirect account of vocabulary comprehension. The MCDI, originally developed in English, has good reliability and validity and has been adapted to over 50 languages and dialects, including Spanish (Jackson-Maldonado et al., 2003; Fenson et al., 2006). The Spanish adaptation, the IDHC was also used in the present study. The Words and Gestures inventory was used at both 18 and 24 months of age as a parent report measure of receptive vocabulary to complement the behavioral assessment (the CCT). In this way, we obtain both a parent report and a child performance measure of the same underlying construct: within-language receptive vocabulary size in English and Spanish. The inventory has 396 words and covers 19 different categories (e.g., animal names, vehicles, toys, food and drink, clothing, body parts, action words, household objects, descriptive words, pronouns, etc.). As a point of comparison with the CCT, the MCDI, and IDHC each cover approximately 229 nouns, 55 verbs, and 37 adjectives among other word types (prepositions, quantifiers, etc.). In addition, the breadth of the MCDI provides two additional measures: Total Conceptual Vocabulary (TCV) and TEs. TEs were calculated as synonyms across English and Spanish with cognates and semi-cognates included. TCV was calculated by summing vocabulary size across languages and subtracting known TEs. TCV indexes the number of lexicalized concepts across languages (Pearson et al., 1993, 1995; Marchman and Martínez-Sussmann, 2002; Conboy and Thal, 2006; Marchman et al., 2010).

#### Intermodal Preferential Looking Priming Task

We used an adaptation of the Intermodal Preferential Looking Task (Golinkoff et al., 1987), which has previously been used to investigate lexical-semantic priming in young toddlers (Arias-Trejo and Plunkett, 2009; Styles and Plunkett, 2009; Singh, 2014). A total of 108 English and Spanish words known by 60% or more of 18-months-old were chosen based on MCDI and IDHC norms (Fenson et al., 2007; Frank et al., 2017). One third of these words served as auditory primes, another third as targets, and a final third as distractor images. In the current study, semantic relatedness is determined by the cosine value between two words: a calculation of the feature overlap between pairs of words based on adult norms (Buchanan et al., 2013). Features can be physical, functional, and categorical. A cosine of 0 represents no semantic overlap between two words, whereas 1 represents complete overlap. Previous work has demonstrated semantic priming in children at these same ages using English adult norms and has extended English-speaking norms to other languages (e.g., Mandarin; Singh, 2014). The lexical items in the current study are highly imageable and early occurring nouns known by the majority of young English and Spanish speakers.

Participants were presented with Related and Unrelated trial types. On related trials, the prime word was semantically related to the target word (e.g., banana and apple). Primes and targets were highly related and had an average cosine value of 0.32 (*SD* = 0.11, range = 0.14–0.55). Unrelated trials presented pairs of unrelated words with a cosine value of 0 (e.g., pillow and apple, “I saw a pillow… apple!”).

The experiment consisted of four blocks: Spanish prime words to Spanish targets (Spanish-Spanish), Spanish prime words to English targets (Spanish-English), English prime words to English targets (English-English), and English primes to Spanish targets (English-Spanish). Each block consisted of three related trials and three unrelated trials. The order of blocks was counterbalanced across participants and trial presentation was pseudo-randomized within blocks such that no more than two trials of the same type were presented consecutively. Side of target presentation was also counterbalanced across participants. Target and distractor pairs were yoked (so as to always appear together), and were semantically unrelated (cosine value = 0). The target and distractor images were non-contiguous (approximately 10 cm apart) to insure accurate location of gaze on the screen (Holmqvist et al., 2011). Each yoked target-distractor pair of images appeared equally across trial types (related and unrelated) to insure that differences in performance across trial types could not be attributed to unanticipated differences in image salience between the target and distractor. Children saw each target and distractor pair only once. Target and distractor images appeared with equal frequency across trial types, though only the target image was named. Similarly, prime words were counterbalanced and appeared equally across trial types. A female native speaker of English and Spanish produced all auditory stimuli in infant-directed speech. Each word and sentence frame was recorded in isolation.

**Figure 2:**
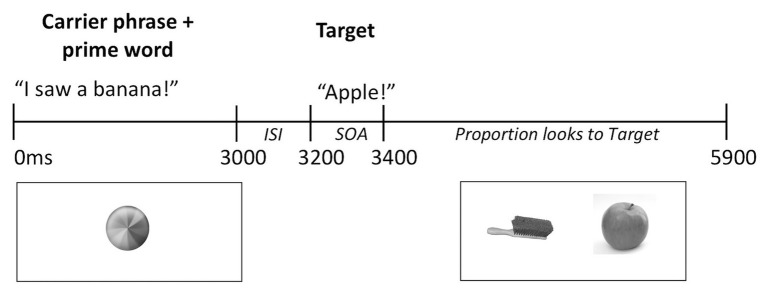
Trial sequence with example. SOA: Stimulus Onset Asynchrony, ISI: Inter-stimulus Interval.

Trials began with the presentation of a carrier phrase ending with the prime word that was either semantically related or unrelated to the target word. An attention-getter (i.e., a spinning water wheel, Luche et al., 2015, see Figure 2) appeared on screen for a total of 1,000 ms during the presentation of the carrier phrase and prime word. Two hundred milliseconds after the offset of the prime word the auditory target word was presented in isolation. Two hundred milliseconds after the onset of the target word, the yoked target and distractor images were presented for a total of 2,500 ms. A short prime-target inter-stimulus interval (ISI) and stimulus-onset asynchrony (SOA) of 200 ms each have been previously used with infants and toddlers (e.g., Arias-Trejo and Plunkett, 2009; Styles and Plunkett, 2009, 2011) and correspond to parameters for lexical priming in adults.

Children were tested across two separate visits: an English and Spanish visit, respectively. The language of the visit determined the language of the primes and was counterbalanced across participants. Within each visit, the within-language priming block preceded the cross-language block (e.g., English – English priming block followed by English – Spanish or Spanish – Spanish followed by Spanish – English). This was preferred over counterbalancing all blocks because it minimized possible task order effects based on language dominance as seen in adult bilinguals (e.g., Misra et al., 2012). In addition, we designed the task such that within-language priming preceded cross-language priming to make cross-language priming as conservative as possible. Theoretically, the preceding language block would make the first language presented more active than the other. Thus, if priming from one language another occurs despite heightened activation to the prime language it suggests that a robust cross-language priming effect is in place early in the second year of life. Lastly, the within-language priming block preceded the cross-language priming block to ensure that direct comparison to monolingual findings was possible given the theoretical motivation for the present study.

##### Data Processing

Several data processing procedures were applied to the eye-tracking data so as to maximize its accuracy based on published best practice guidelines. A median moving window filter was applied to the raw eye-tracking data in which data was chunked in 100 ms windows and a single median was calculated (Wass et al., 2014) even when data for only a single eye was available (Saez de Urabain et al., 2015). Gaze data were interpolated when a gap of no more than 150 ms was encountered (Wass et al., 2014; Saez de Urabain et al., 2015). Interpolation was achieved by creating a continuous trajectory through scaling gaze samples before and after the gap (Olsen, 2012). Trials in which toddlers fixated on the target and distractor less than 25% of the trial were removed. In addition, trials in which toddlers fixated only the target or only the distractor were excluded to increase the probability that the data reflected active lexical-sematic processing (e.g., Mani and Plunkett, 2010). The *eyetracking R* package (Dink and Ferguson, 2015) in RStudio (RStudio R Team, 2015) was used to measure proportion looking by calculating total looking to the target divided by the sum of total looking to the target and distractor.

### Procedure

After a short warm-up play period between the toddler and the experimenter, children and their caregivers were escorted to a dimly-lit room, which houses a touch-sensitive monitor on which we administered the CCT. Next, children and their parents moved to a second room to complete the IPL task. For the IPL procedure, toddlers sat approximately 25 in away from a 27'' Dell monitor with a 1920×1080 resolution. A Tobii X120 recorded eye gaze and was placed 3 in below and 5 in in front of the monitor. Auditory stimuli were presented through Dell AX210 multimedia stereo audio speakers. Following the IPL task, caregivers were asked to complete the parent report of vocabulary on the MCDI and IDHC.

## Results

### Vocabulary

Children demonstrated increased receptive vocabulary in English and Spanish on the CCT behavioral measure from 18 months (English CCT: *M* = 14.16, *range* = 4–25; Spanish CCT: *M* = 12.07, *range* = 4–21) to 24 months of age (English CCT: *M* = 23.08, *range* = 2–35; Spanish CCT: *M* = 19.35, *range* = 6–26) across English and Spanish. Parent reports of receptive vocabulary on the MCDI and IDHC were relatively balanced across English and Spanish at 18 months (MCDI: *M* = 190.71, *range* = 22–373; IDHC: *M* = 180.43, *range* = 26–355) and 24 months of age (MCDI: *M* = 285.5, *range* = 59–396; IDHC: *M* = 226.0, *range* = 14–396).

In addition, we calculated measures of cross-language knowledge. Specifically, comparisons of the English and Spanish MCDI and IDHC yielded measures of TEs and TCV, both of which increased from 18 to 24 months. In general, TEs made up a significant portion of children’s vocabulary (18 months: *M* = 113.13, *range* = 13–230; 24 months: *M* = 162.10, *range* = 13–307). Children’s non-dominant language was made up of a larger proportion of TEs (18 months: *M* = 60%, 24 moths: *M* = 66%) relative to the dominant language (18 months: *M* = 51%, 24 months: *M* = 49%) at both 18 and 24 months of age. Further, TCV increased by approximately 25% over the 6 month period (18 months: *M* = 279.19, *range* = 70–428; 24 months: *M* = 366.59, *range* = 70–491).

### Lexical-Semantic Priming at 18 and 24 months

#### Total Looks

**Figure 3:**
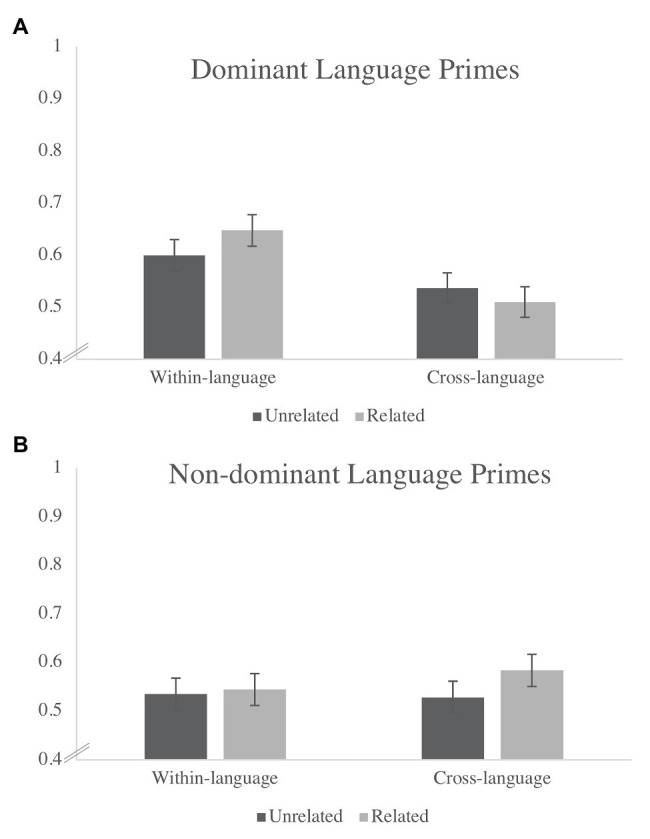
Average proportion of time spent looking at the target across related and unrelated trials at 18 months when primes were in the **(A)** dominant language compared to the **(B)** non-dominant language across within- and cross-language blocks.

**Figure 4:**
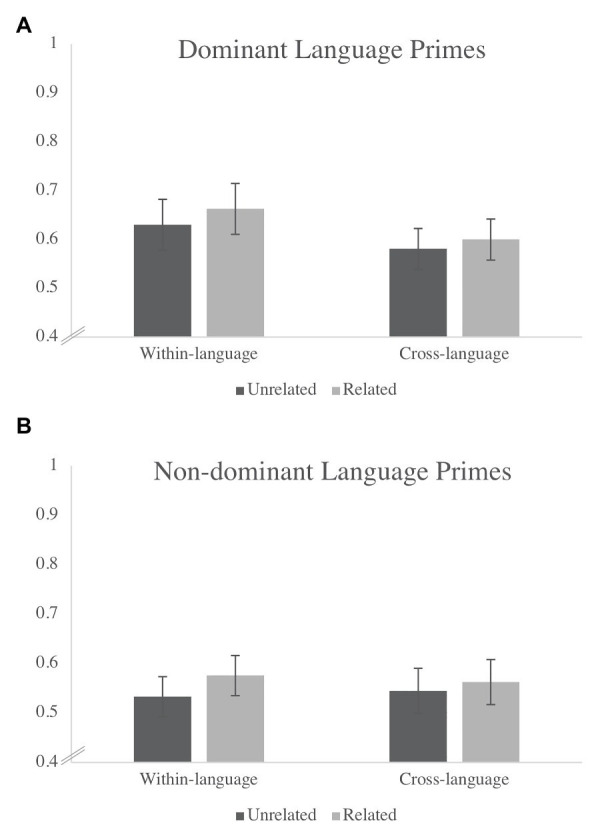
Average proportion of time spent looking at the target across related and unrelated trials at 24 months when primes were in the **(A)** dominant language compared to the **(B)** non-dominant language across within- and cross-language blocks.

The first step in the analysis was to replicate previous coarse-grained findings by aggregating over the entire time window of interest and asking whether proportion of total looks differed significantly as a function of language (dominant language vs. non-dominant language, based on exposure) and trial types (semantically related vs. unrelated) at 18 and 24 months, respectively. Thus, a repeated-measures ANOVA was conducted to examine whether proportion of total looks differed as a function of three repeated factors: Language (Dominant or Non-Dominant), Prime Type (Within‐ or Cross-Language), and Trial Type (Related or Unrelated). At 18 months, there was a significant main effect of Language [*F*(1, 28) = 6.25, *p* = 0.02, *η_p_*^2^ = 0.19] and a significant Language X Prime Type interaction [*F*(1, 28) = 4.6, *p* = 0.04, *η_p_*^2^ = 0.14]. As seen in Figure 3, the interaction reflects the fact that children evinced the longest looking times in the within-language condition in the dominant language. There were no other significant effects (all *p*s > 0.08) and, in particular, no effect of Trial Type (*p* = 0.31). Yet, by 24 months of age, results revealed a significant main effect of Trial Type [*F*(1, 16) = 5.73, *p* = 0.03, *η_p_*^2^ = 0.26, see Figure 4] indicating a difference in looking time between related and unrelated trials by the end of the second year. There were no other significant effects at 24 months (all *p*s > 0.23).

#### Looks Over Time

**Figure 5:**
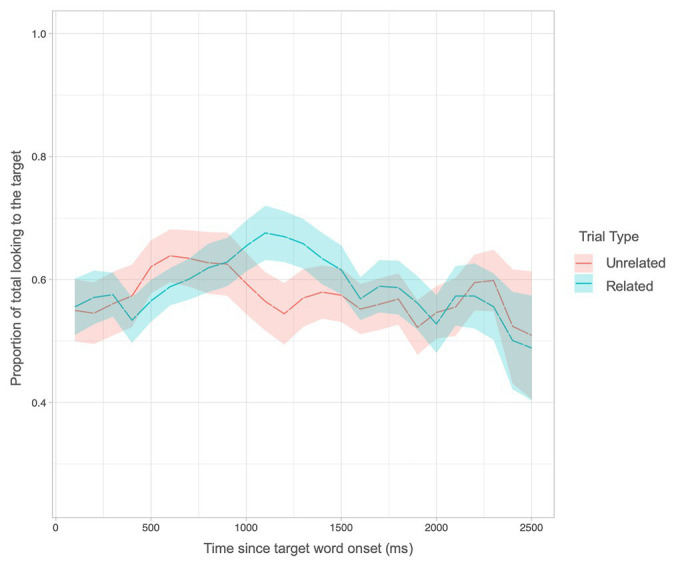
Proportion of looks to the target for related and unrelated trials over time at 18 months.

**Figure 6:**
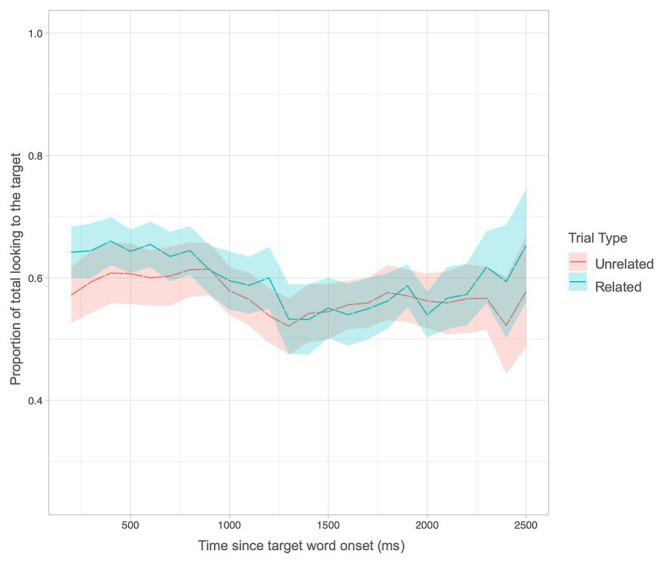
Proportion of looks to the target for related and unrelated trials over time at 24 months.

Next, we asked whether looking times differed over the course of the trial using a growth curve analysis. This analysis affords a fine-grained, millisecond-by-millisecond, view of children’s changes in looking behavior. At 18 months, there was a significant quadratic term indicating a Trial Type interaction (LRT = 5.89, *p* = 0.015). This interaction suggests that looking patterns differed over the course of the trial for Related and Unrelated trials. As can be seen in Figure 5, children spent more time looking to the target object on related versus unrelated trials between 1,000 and 1,500 ms, a relatively short period of time late in the time window of interest. At 24 months, results revealed a main effect of Trial Type (LRT = 3.9, *p* = 0.04) consistent with the ANOVA results. However, there was no interaction with any of the polynomial change terms, indicating that looking times did not vary over time. Rather, as can be seen in Figure 6, looking times to the target for related trials were consistently above unrelated trials throughout much of the time window. Thus, time-sensitive analyses reveal sensitivity to semantic relatedness between word pairs as early as 18 months.

### Associations Between Vocabulary and Lexical-Semantic Priming

Last, we examined the relation between vocabulary size and lexical-semantic priming. To calculate the size of the prime effect, the difference between children’s average proportion of looks to the target on semantically related and unrelated trials was calculated. Recall that we expected children to evince longer looking to semantically related trials relative to unrelated trials if they are sensitive to the semantic relation between words. As such, the numerical difference in looking behavior between the two trial types provides a measure of the prime effect and an index of children’s sensitivity to semantic associations between word pairs within and across languages. The next set of analyses therefore asked whether the degree of the prime effect was associated with several measures of lexical knowledge: within-language vocabulary, TEs, and TCV.

We conducted a series of repeated measures ANCOVAs to analyze the effect of measures of vocabulary on looking times across trials. Age (18 vs. 24 months), Prime Type (Within‐ or Cross-language), and Language (Dominant or Non-Dominant) were entered as factors, the vocabulary measure of interest was entered as a covariate, and proportion looks to the target was the dependent measure.

First, we examined whether within-language receptive vocabulary would predict lexical-semantic priming effects. Recall that we had two measures of receptive vocabulary: a behavioral task (CCT) and parent report (MCDI). Across both measures, there were no significant main effects or interactions, though a marginal CCT X Wave X Language interaction (*p* = 0.06) was evinced. This suggests that receptive vocabulary in one language does not predict the size of the prime effect within or across languages.

Next, we examined cross-language vocabulary measures. Here, we assessed TEs and TCV. We observed a significant TEs X Prime Type interaction [*F*(1, 68) = 4.82, *p* = 0.03]. Follow-up analyses indicated that TEs were positively correlated with the size of the prime effect in *within*-language trials, although this association was marginal [*r*(30) = 0.27, *p* = 0.05]. Conversely, there was no association between TEs and the prime effect in *cross*-language trials. Similarly, the second model revealed a significant TCV X Prime Type X Wave X Language interaction [*F*(1, 68) = 4.16, *p* = 0.04]. Follow-up analyses yielded a pattern similar to TEs, though language dominance influenced the pattern over time. Specifically, TCV was marginally correlated with the prime effect in within-language trials for the dominant language at 18 months [*r*(30) = 0.44, *p* = 0.07], and for the non-dominant language at 24 months [*r*(30) = 0.72, *p* = 0.07].

## Discussion

The present study sought to examine lexical-semantic development longitudinally at 18 and 24 months of age in Spanish-English bilinguals and to investigate the role of vocabulary knowledge on the degree of the lexical-semantic priming within and across languages. Specifically, we asked whether lexical-semantic priming emerges at 18 or 24 months of age in bilinguals and whether within-language vocabulary size, TCV size, and number of known TEs were significant predictors of children’s lexical-semantic processing.

With respect to the emergence of lexical-semantic priming, results revealed a pattern of development similar to that documented in monolinguals. As reviewed previously, findings within the monolingual literature suggest a fragile emergence of lexical-semantic priming, such that priming was evinced at 21 and 24 months of age but not at 18 months (Arias-Trejo and Plunkett, 2009, 2013; Styles and Plunkett, 2009). Indeed, traditional ANOVA analyses in the present study replicated these findings, showing a significant difference in looking time between related and unrelated trials at 24 but not 18 months of age. That is, given adequate power, coarse-grained analyses that collapse over the time window of the priming trials, reveal a significant difference between semantically related and unrelated trials at 24, but not at 18, months in bilingual children consistent with previous findings in monolingual children. Nevertheless, the growth curve analyses examining gaze as a function of time revealed that the pattern of looking behavior indeed differed across the time window at 18 months. Visual inspection of looking times throughout the time window show that children’s looking time to the target object differed between semantically related and unrelated trials but only later in the trial period, between 1,000 and 1,500 ms at 18 months of age. Conversely, by 24 months, the difference in looking time as a function of semantic relatedness was relatively consistent throughout the entire time window. This provides evidence for developmental changes occurring from 18 to 24 months that support increasing efficiency in processing the lexical-semantics of spoken words.

What do these findings reveal about the development of lexical-semantic processing? The similarity in the pattern of results with previous monolingual research is consistent with a large body of work demonstrating that dual and single language exposure leads to a similar timetable for the acquisition of early words and for the development of semantic networks in some cases. Indeed, as reviewed previously, important language milestones, such as babbling, first word production, and word combinations are all met at the same time for children learning one or two languages (e.g., Pearson et al., 1993; Pearson and Fernández, 1994). Similarly, processing measures demonstrate similar development in single and dual language contexts: monolingual and bilingual children employ similar word learning strategies (Byers-Heinlein et al., 2013). Speed of lexical access is also similar between monolingual and bilingual toddlers at 16 and 22 months of age (Legacy et al., 2015, 2016; De Anda et al., 2018). Thus, the present findings add to this body of work by demonstrating that early bilingual language learners demonstrate an emerging lexical-semantic system that develops incrementally throughout the second year in a timetable comparable to their monolingual peers (Arias-Trejo and Plunkett, 2009, 2013; Styles and Plunkett, 2009; *Removed for blinding*, in review).

Despite the similarities between monolinguals and bilinguals in the emergence of lexical-semantic priming, there are unique circumstances in dual language learners that we examined further in the present study. Recall that the present study sought to examine the influence of vocabulary knowledge in a dual language context in Spanish-English learners by comparing within‐ and cross-language vocabulary measures. Measures of within-language receptive vocabulary size did not reliably predict the lexical-semantic prime effect across 18 and 24 months of age. This suggests that the lexical breadth in each language is not associated with lexical-semantic processing within and across languages. Conversely, measures that account for the entirety of lexical knowledge across languages, such as TEs and TCV, did evince significant effects. TEs were positively associated with the size of the prime effect at 18 and 24 months of age, such that children with more TEs across languages showed greater differences in looking behavior to semantically unrelated and related trials for within-language word pairs. Similarly, the size of children’s TCV across languages was positively associated with the degree of lexical-semantic priming also in within-language contexts only. Further, this association seemed to change over time, such that it was strongest for lexical-semantic processing in the dominant language at 18 months and in the non-dominant language by 24 months. This once more suggests that lexical-semantic skill exhibits an incremental development across the second year, such that as processing in the non-dominant becomes more robust it increasingly relies on cross-language lexical knowledge.

What explains the differences in findings between TEs and TCV and the observed developmental changes? As shown in Figure 1, each measure captures different constructs of cross-language lexical knowledge that are unique to children learning more than one language. TEs offer a relatively precise overlap between lexical and semantic representations. TCV conversely is a measure of the number of concepts that have been lexicalized, and the majority of these lexical items are in the dominant language. The findings suggest that, by the end of the second year, the non-dominant language becomes increasingly reliant on this lexical space, such that a greater conceptual vocabulary in both languages supports stronger priming effects at 24 months. Indeed, recall that the proportion of known TEs increased from 18 to 24 months in the non-dominant language, whereas this proportion stayed relatively stable in the dominant language. This suggests that children were more likely to learn a TE if the word was first acquired in the dominant language and this propensity increased over the 6-month period. Indeed, learning a word in one language facilitates its acquisition in a second language in bilingual children between 6 months and 7 years of age (Bilson et al., 2015). Further, bilingual children exhibit an overall preference for learning words with more associative cues, a word learning strategy that is amplified in the context of TEs. Compared to their monolingual peers, bilingual children learn words in English in a different order. Together the pattern of findings suggests that bilingual children are leveraging cross-language word knowledge to support the non-dominant language. This may explain why TCV shows a stronger association with lexical-semantic priming effects in the non-dominant language over this same time period. That is, the lexical-semantic overlap in the non-dominant language increases over time making the priming effect emerge in the weaker language by the end of the second year.

The present findings add to a growing body of evidence showing that the cross-language associations in the lexical-semantic system are influenced by dominance patterns. For example, in a recent study, vocabulary size in the dominant language supported the speed of lexical retrieval in the non-dominant language at 16 and 22 months of age in Spanish-English bilinguals (De Anda et al., 2018). Similarly, Marchman et al. (2010) observed differences in speed of spoken word processing based on language dominance, such that by 30 months bilinguals showed faster word recognition in the dominant vs. the non-dominant language. Distinct patterns of temporal and spatial neural activation are also observed between the dominant and non-dominant language and these differences in neural processing vary as a function of TCV (Conboy and Mills, 2006). Further, Singh (2014) also showed dominance effects in lexical-semantic priming in English-Mandarin toddlers at 30 months. However, by 30 months, bilinguals demonstrated priming effects only when the prime word was in the dominant language. Though this effect was not observed in the 18‐ and 24-month-old Spanish-English learners observed here, differences may be explained by several factors known to influence lexical acquisition. For example, Spanish and English are much more similar than Mandarin and English, thereby influencing patterns of cross-language processing skill and knowledge. Further, as we have shown in the present study, 6 months of development can lead to large differences in children’s lexical processing skills. Indeed, vocabulary growth differs across language pairs (Floccia et al., 2018), age groups, phonological and semantic domains, and age of acquisition (Angulo-Chavira and Arias-Trejo, 2017).

### Limitations and Future Directions

This study is one of the first to examine lexical-semantic priming longitudinally in bilinguals during the second year, a critical time period in lexical development. Given the dearth of studies in this area there remain many unanswered questions that limit our full understanding of the emergence of lexical-semantic organization in dual language contexts. For example, the present study examined a specific pair of languages that are highly similar (i.e., English and Spanish) whereas other studies have examined less similar languages (i.e., English and Mandarin; Singh, 2014). It is possible that differences in the amount of semantic overlap between languages may influence the quantity, quality, and development of lexical-semantic associations that are formed in early life. This could contribute to group and individual differences alike. Given the findings of the present study, languages with less semantic overlap than Spanish and English could lead to fewer TEs in the early lexicons of children. Less overlap may lead to less robust cross-language lexical-semantic associations. Without the similar structure of a second language, there is less of a need to leverage cross-language associations, meaning that the findings in Spanish and English may not be universal. Under such conditions, it may be that children make use of within-language information more consistently, or perhaps continue to use cross-language information but to a lesser degree than children with a greater number of TEs. Similarly, in language pairs with limited phonological and morphological overlap, it may be more difficult to develop cross-language associations to build lexical-semantic structure. What is likely to be universal is that the degree of overlap in language content, function, and form may facilitate generalization across languages as a general principle. A recent investigation of vocabulary size in toddlers suggests that linguistically similar language contexts promote larger vocabularies in 24-months-old (Floccia et al., 2018). Recall that a growing body of research has also shown a rich interaction between phonology and semantics across languages in German-English learners (Von Holzen and Mani, 2012). Together these findings suggest that all of the domains of language (phonology, semantics, morphology, syntax, pragmatics, etc.) play a role in determining the linguistic distance across languages and its potential influence on lexical-semantic organization in multilingual contexts. Future studies should consider examining several domains simultaneously to further elucidate word recognition processes.

In addition, the priming adaptation of the IPL paradigm has allowed researchers to examine word-to-word semantic processing, but it tells us little about semantic processing in real-world language input. Indeed, language occurs in multi-word utterances and not in single words. As such, future studies should examine how lexical-semantic processing occurs at the sentence level. In this way, we can begin to understand how lexical-semantics interfaces with grammar, phonology, prosody, and the semantics of other nearby words. In addition, in the present study, within‐ and cross-language priming blocks were not counterbalanced in the interest of comparing to extant monolingual literature (within-language blocks always preceded cross-language blocks). Thus, it is possible that cross-language priming may have been attenuated because of the preceding single-language context. Indeed, an open question for future research is whether setting up a language context indeed influences subsequent semantic processing within and across languages in young children.

### Conclusion

The present study investigated whether lexical-semantic priming emerges as early as 18 or 24 months of age in Spanish-English bilinguals and whether vocabulary knowledge within or across languages predicts children’s lexical-semantic processing. The results indicate a remarkably similar pattern of development between monolingual and bilingual children, such that lexical-semantic development begins slowly at 18 months and becomes more robust by the end of the second year. Specifically, coarse proportion looking analyses show a lexical-semantic priming effect at 24 but not 18 months of age, whereas fine-grained growth curve analyses reveal that semantic organization begins as early as 18 months of age in bilingual children. This manuscript reveals the earliest emergence of lexical-semantic organization at 18 months and its more mature instantiation at 24 months of age. Further, measures of lexical knowledge that take into account both languages are stronger predictors of children’s lexical-semantic priming abilities than within-language measures. Together these findings provide support for the conclusion that important language milestones are met at the same time across single and dual language learners, though unique cross-language consequences of dual language exposure lead to complex associations between lexical knowledge and semantic processing skill.

## Data Availability Statement

The datasets generated for this study are available on request to the corresponding author.

## Ethics Statement

The studies involving human participants were reviewed and approved by San Diego State University Institutional Review Board. Written informed consent to participate in this study was provided by the participants’ legal guardian/next of kin.

## Author Contributions

SD designed and collected the data for the present study with supervision from MF. SD took the lead in drafting all parts of the manuscript and MF provided critical feedback to help shape the research, analysis, and dissemination of findings. Both the authors contributed to the article and approved the submitted version.

### Conflict of Interest

The authors declare that the research was conducted in the absence of any commercial or financial relationships that could be construed as a potential conflict of interest.
